# Emerging and re‐emerging fungal threats in Africa

**DOI:** 10.1111/pim.12953

**Published:** 2022-10-17

**Authors:** Rachael Dangarembizi, Sean Wasserman, Jennifer Claire Hoving

**Affiliations:** ^1^ Division of Physiological Sciences, Department of Human Biology, Faculty of Health Sciences University of Cape Town Cape Town South Africa; ^2^ Neuroscience Institute, Faculty of Health Sciences University of Cape Town, Groote Schuur Hospital Cape Town South Africa; ^3^ CMM AFRICA Medical Mycology Research Unit, Institute of Infectious Disease and Molecular Medicine, Faculty of Health Sciences University of Cape Town Cape Town South Africa; ^4^ Wellcome Centre for Infectious Diseases Research in Africa, Institute of Infectious Diseases and Molecular Medicine University of Cape Town Cape Town South Africa; ^5^ Division of Infectious Diseases and HIV Medicine, Department of Medicine University of Cape Town, Groote Schuur Hospital Cape Town South Africa; ^6^ Division of Immunology, Department of Pathology, Faculty of Health Sciences University of Cape Town Cape Town South Africa

**Keywords:** dimorphic fungal pathogens, emerging fungi, fungal pathogens, opportunistic fungal infections

## Abstract

The emergence of deadly fungal infections in Africa is primarily driven by a disproportionately high burden of human immunodeficiency virus (HIV) infections, lack of access to quality health care, and the unavailability of effective antifungal drugs. Immunocompromised people in Africa are therefore at high risk of infection from opportunistic fungal pathogens such as *Cryptococcus neoformans* and *Pneumocystis jirovecii*, which are associated with high morbidity, mortality, and related socioeconomic impacts. Other emerging fungal threats include *Emergomyces* spp., *Histoplasma* spp., *Blastomyces* spp., and healthcare‐associated multi‐drug resistant *Candida auris*. Socioeconomic development and the Covid‐19 pandemic may influence shifts in epidemiology of invasive fungal diseases on the continent. This review discusses the epidemiology, clinical manifestations, and current management strategies available for these emerging fungal diseases in Africa. We also discuss gaps in knowledge, policy, and research to inform future efforts at managing these fungal threats.

## 1 INTRODUCTION

The human body is a thermally restrictive environment to the survival of fungi, most of which cannot survive at our body temperature of 37°C.^1^, ^2^ Consequently, only about 0.2% of the 1.5 million fungal species known to exist since over 1.6 million years ago, have the potential to cause human disease.^3^ For much of human history, fungal pathogens were not a threat to human health but climate change and changing environmental pressures have been attributed as potential drivers of the evolution and emergence of new thermotolerant, potentially pathogenic fungal species.^4^, ^5^, ^6^ Additionally, the advancement of medical sciences (haematopoietic stem cell and solid organ transplants, antibiotics, immunosuppressant therapy), coupled with the emergence of immunosuppressive diseases at the turn of the twentieth century, led to the rise of highly fatal opportunistic fungal infections. There are several reasons why the African population is particularly vulnerable to emerging fungal threats. First, Sub‐Saharan Africa bears the greatest burden (almost 70%) of human immunodeficiency virus (HIV) infections globally^7^ and has a substantial proportion of immunosuppressed individuals, who are vulnerable to opportunistic infections. Second, health systems in Africa rely mostly on private health care (out‐of‐pocket payments from patients) and donor funding with average government health expenditure (as a proportion of general expenditure) in Sub‐Saharan Africa sitting at a meagre 7.2%.^8^ With almost 500 million people living in extreme poverty (<$1.90 purchasing power parities [ppp]/day),^9^, ^10^ this means that more than one third of the African population cannot afford basic health care, hospitalization, or antifungal drugs. Other challenges affecting the continent include challenges with antifungal drug availability and registration policies, and a lack of consistent power supply. There is also a lack of skills and adequate infrastructure in primary care facilities thus high‐level care is restricted to referral hospitals which are usually in the cities meaning that most patients present to referral hospitals with advanced disease.

The aim of this review is to discuss disease‐causing fungi that have remained problematic and/or have arisen on the African continent over the past 30 years. We review current literature on the epidemiology, pathogenesis, and clinical manifestations of these emerging and re‐emerging fungal diseases, highlighting gaps in current knowledge and directions for future research. The review will focus on fungal pathogens that are associated with high morbidity and mortality in Africa, including *Cryptococcus* spp. and *Pneumocystis* spp. both opportunistic fungal infections affecting immunodeficient people living with or without HIV/AIDS. We also discuss other emerging fungal pathogens of concern in Africa including dimorphic fungi such as *Histoplasma* spp., *Emergomyces* spp. and *Blastomyces* spp. *Candida* spp. may be an emerging threat due to increasing drug resistance and rise of non‐albicans species such as *Candida auris* and *Candida parapsilosis*. We consider how socioeconomic development and the Covid‐19 pandemic may lead to shifts in epidemiology of invasive fungal diseases on the continent.

## 2 *CRYPTOCOCCUS* SPP.

**FIGURE 1:**
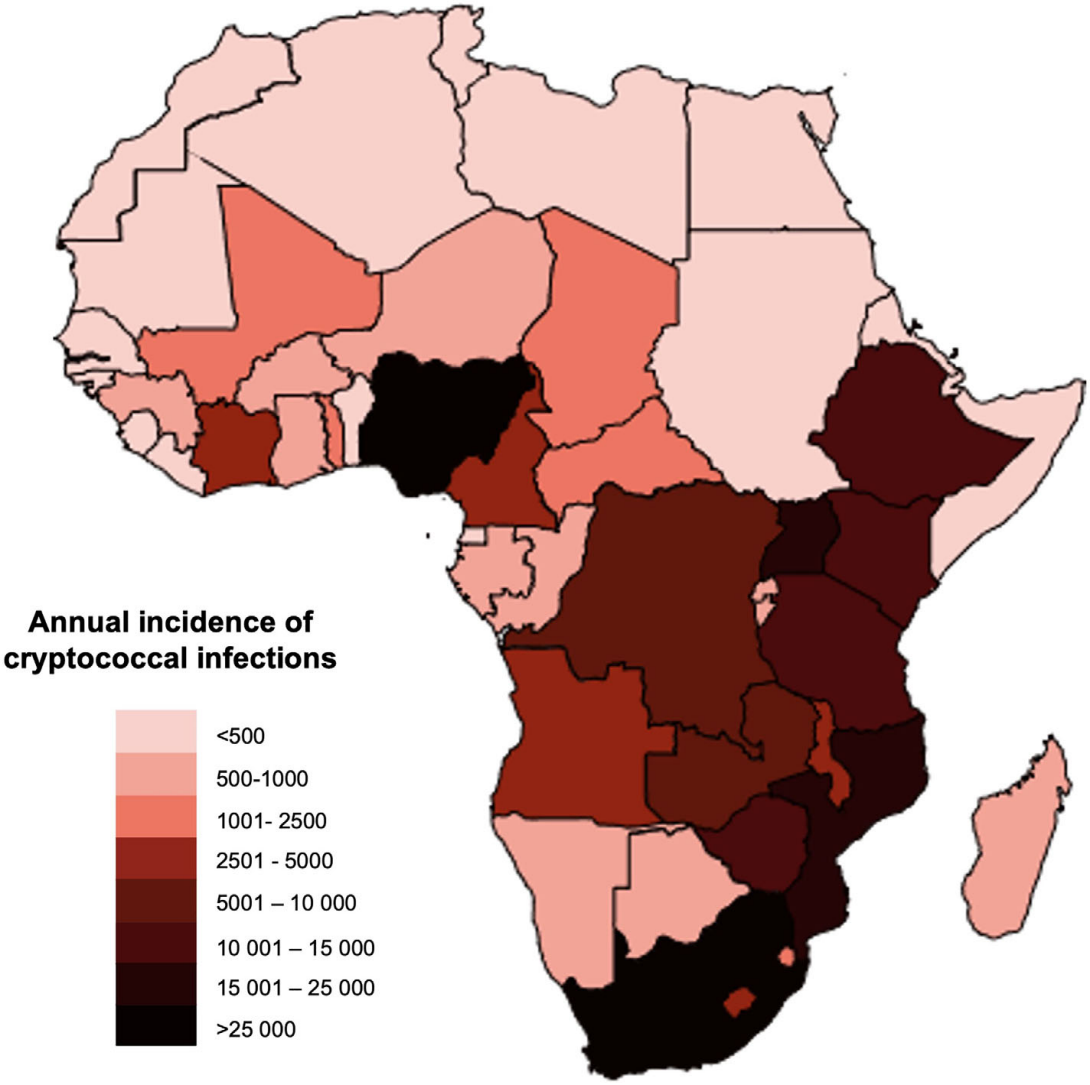
Annual incidence of cryptococcal infections in Africa *(modified from* ^13^)

*Cryptococcus* re‐emerged as a fatal opportunistic pathogen during the HIV/AIDS pandemic in the late 1980s.^10^ Although already known to be associated with immunosuppressed individuals in scattered cases for over 100 years, cryptococcal meningitis infections rose to almost reach the million mark at the peak of the HIV pandemic.^11^ Introduction of antiretroviral therapy (ART) significantly reduced incidence in high income countries, but Africa continues to battle a large HIV‐associated epidemic of cryptococcal disease due to challenges of access and adherence to ART.^12^ Cryptococcal infection remains a major cause of meningitis in Africa, ahead of bacterial and tuberculous meningitis, with 10–12‐week mortality rates of between 40–70%.^11^, ^13^, ^14^ Sub‐Saharan Africa currently accounts for 73% of the estimated 223,100 global cryptococcal meningitis cases, and 75% of the estimated 181,000 deaths^13^ (see Figure 1 for the annual incidence of cryptococcal infections in Africa).

Cryptococcal meningitis is caused by the basidiomycetes *Cryptococcus neoformans* and *Cryptococcus gattii*; the only two clinically significant species of a 36‐member genus, which are commonly found near decaying wood, pigeon guano, and in contaminated soil.^15^, ^16^ *C. neoformans* is responsible for more than 90% of all infections in Africa.^15^, ^16^ *C. gattii* has principally been associated with infection in people without overt immune deficiencies, although HIV‐related *C. gattii* infections have also been reported.^17^, ^18^ Cryptococcal infection occurs via inhalation of yeast cells or spores, which for most people, occurs early in life. For immunocompetent individuals, the initial pulmonary infection is often cleared by the host immune system, but fungal cells may lie dormant for years with no clinical symptoms experienced by the host. Reactivation of the latent infection to its life‐threatening form occurs when the host immune system is suppressed, mainly through iatrogenic causes or HIV infection. CM incidence is much lower in children living with HIV (annual incidence of 10–50 cases/1000000) as compared to their adult counterparts (120 cases/100000).^19^, ^20^

In the lungs, cryptococcal antigens are recognized by alveolar macrophages and dendritic cells; keys cells of the innate immune system which phagocytose the fungus and activate the adaptive immune response.^21^ Importantly, antigen presentation by dendritic cells stimulates naive T cells to differentiate into three subtypes of mature helper T (Th) cells (Th1, Th2 or Th17) depending on the antigen recognized and cytokine milieu.^22^, ^23^, ^24^ Th1 and Th17 responses to cryptococcal infection are associated with the release of the proinflammatory cytokines interferon (IFN)‐γ and interleukin (IL)‐17, respectively, which promote fungal clearance. Th2 responses are typically non‐protective against cryptococcal infection and are associated with the release of the inappropriate cytokines IL‐4, IL‐5 and IL‐13, which promote fungal persistence, dissemination and worsened pathology.^23^ Using a repertoire of virulence factors (mainly the capsular polysaccharide glucuronoxylomannan [GXM]), *Cryptococcus* can skew the polarization of the adaptive immune response towards the more permissive and non‐protective Th2‐type response.^25^ This immune unresponsiveness then allows the fungus to proliferate and disseminate to extra‐pulmonary structures, with a predilection for the brain. Cryptococcal neurotropism is believed to be driven by the abundance of phenolic compounds in neural tissue including neurotransmitters such as dopamine, serotonin and norepinephrine which serve as precursors for the synthesis of melanin by the enzyme laccase, a key virulence factor for the fungal pathogen (Zhu and Williamson, 2004; Colombo and Rodrigues, 2015).

Disseminated cryptococcal cells cross the blood brain barrier (BBB) using several possible ways; (1) a Trojan horse mechanism, by which fungal cells are ferried by infected peripheral monocytes,^26^ (2) a transcellular transport mechanism, by which fungal cells enter endothelial cells and cross into the brain^27^ and (3) a paracellular transport mechanism, by which fungal cells use virulence factors (such as urease) to disrupt the integrity of BBB tight junctions to facilitate their passage into the brain parenchyma.^28^ Presence of cryptococcal cells in the central nervous system (CNS) is associated with meningoencephalitis which is the most fatal form of the disease and the major cause of death. The neuroimmune response to cryptococcal presence is not very well defined and remains a gap in our knowledge of the pathophysiology of the disease, especially at the molecular level (see^29^ for a review). However, previous research suggests that central defences systems to C*ryptococcus* may not be very different from the dichotomous pattern observed in peripheral defences to the fungus in which a pro‐inflammatory response is beneficial, while an anti‐inflammatory response is non‐protective and detrimental.^30^ Survival and mortality following cryptococcal infection seem dependent on the nature of the host's immune response to central infection. A paucity of inflammation in the cerebrospinal fluid (CSF) compartment is associated with decreased fungal clearance, increased fungal burden, increased intracranial pressures, deteriorating neurological signs and higher mortality.^31^, ^32^ On the contrary, patients showing higher levels of IL‐6, IFN‐γ, tumour necrosis factor (TNF)‐α and IL‐8 had higher rates of fungal clearance.^31^, ^33^ Previous research has shown that suppressing the Th2 response by knocking out IL‐4, IL‐4R and IL‐13 in mice increases resistance to cryptococcal infection, with very little infiltration or persistence of fungal cells in the CNS compared to wild type. On the other hand, overexpression of IL‐13 is associated with more severe CNS invasion and the development of large pseudocystic lesions surrounded by yeast‐filled, alternatively activated macrophages.^34^ The administration of pro‐inflammatory cytokines (IL‐12, IL‐18, IFN‐γ and TNF‐α) as adjunctive therapy during cryptococcosis has been associated with better survival of infected mice.^35^, ^36^, ^37^, ^38^, ^39^, ^40^ In two, phase‐II clinical trials, rapid CSF sterilization was reported in HIV patients receiving adjuvant recombinant IFN‐γ 1b.^41^, ^42^ Similarly, negative CSF cultures and complete clinical recovery were reported for a patient with idiopathic CD4+ lymphopenia who received 50 μg of IFN‐γ, three times weekly for four weeks.^43^

A paradoxical, life‐threatening form of cryptococcal meningitis, cryptococcosis‐associated immune reconstitution inflammatory syndrome (C‐IRIS), is reported in 10%–50% of HIV‐positive individuals upon recruitment to ART.^44^, ^45^ C‐IRIS is characterized by a paradoxical worsening of clinical symptoms observed upon recruitment to ART, which results from a hyperinflammatory response to either a pre‐existing (partially treated or residual) cryptococcal infection, or an unmasked and previously subclinical cryptococcal infection.^46^, ^47^, ^48^ The World Health Organization (WHO), therefore, recommends cryptococcal antigen (CrAg) screening before commencing ART. In addition, WHO also recommends CrAg screening for HIV‐infected adults (and adolescents) with CD4+ counts of 100–200 cells/mm^3^ and pre‐emptive fluconazole therapy for all CrAg‐positive individuals before initiation ART to prevent the development of severe cryptococcal disease.^49^ The CrAg lateral flow assay (LFA) a highly sensitive immunochromatographic dipstick test that can be used at point‐of‐care to measure the presence of GXM in serum, plasma, or CSF has changed the landscape of cryptococcal meningitis diagnosis in Africa because it is cheap, does not require laboratory infrastructure and has a good shelf‐life of up to two years with no need for refrigeration.^50^

Current treatment recommendations for clinical cryptococcal meningitis involves three phases: (1) an induction phase on a combination of amphotericin B deoxycholate (AmB‐D) and flucytosine for one week followed by another week on high‐dose (1200 mg daily) fluconazole, (2) a consolidation phase on medium dose (800 mg daily) fluconazole for 8 weeks after induction and (3) a maintenance phase on low dose (200 mg daily) fluconazole for up to twelve months to prevent relapse of infection.^49^ Implementation of these treatment guidelines in Africa is challenging. Firstly, flucytosine is not registered in fifty of the fifty‐four African countries [see full details of antifungal drug availability and registration on https://gaffi.org/antifungal-drug-maps/]; therefore, it is unavailable as an option to administer during the induction phase for most countries. Intravenous AmB‐D administration requires hospitalization and constant monitoring for side effects which include hypokalaemia, nephrotoxicity and anaemia.^51^, ^52^ A recent clinical trial (AMBITION‐cm) conducted across five countries in Sub‐Saharan Africa showed that a single dose of liposomal amphotericin B (L‐AmB) administered in combination with flucytosine and fluconazole during the induction phase was as effective as standard of care but with less side effects.^53^ However, the widespread use of L‐AmB is still hindered by its high cost and unavailability in most countries.^54^ Most patients end up receiving the suboptimal fluconazole monotherapy which has been associated with high rates of mortality.^55^, ^56^ In addition, there have been growing concerns about the emergence of fluconazole‐resistant *cryptococcus* strains which may be partly responsible for a high risk of relapse in cryptococcal meningitis patients.^57^, ^58^, ^59^ In summary, the high mortality due to cryptococcosis in Sub‐Saharan Africa is driven by (1) a lack of access to effective antifungal drugs with most countries unable to offer combination therapy due to the unaffordability, unavailability and lack of registration of flucytosine, (2) a lack of laboratory capacity to safely administer AmB‐D and monitor potential toxicity events and (3) lack of access to affordable and accessible healthcare facilities, which means that patients often present with advanced disease and likewise cannot sustainably continue with the lengthy treatment regimens and follow‐ups.^60^

## 3 PNEUMOCYSTIS JIROVECII

*Pneumocystis jirovecii* is a human specific opportunistic pathogen characterized by a strong tropism for the lungs and airborne host‐to‐host transmission. It causes pneumonia (PCP) in predisposed patients with worldwide distribution. *Pneumocystis* spp. are fascinating in that they were previously thought to be protozoa^61^ and are thought to have evolved together with specific hosts thereby driving obligate biotrophy.^62^ Very little was known about the biology of *Pneumocystis* spp., with the major limitation in that it has never convincingly been cultured. The recently published genome data have revealed many key insights.^63^ Interestingly, *Pneumocystis* has lost many metabolic pathways, but retains those critical to survival in the host environment. This data suggest that *Pneumocystis* scavenges from the host for survival and has lost components that are important drug targets, such as ergosterol (the target of azoles and amphotericin B) and the trophic form has no β‐glucan (the target of echinocandins).^63^ PCP is an AIDS‐defining illness and lead to the discovery of HIV in the eighties. Since the introduction of ART and anti‐*Pneumocystis* prophylaxis, PCP numbers were significantly reduced in developed countries. However, in recent years, evidence from the UK, USA and Europe suggests that PCP is re‐emerging.^64^, ^65^, ^66^, ^67^ This is due to the increased number of people requiring immunosuppressive therapy.

In developing countries, particularly in Sub‐Saharan Africa, the roll out of ART was complicated and, therefore, the rate of PCP is high. PCP prevalence is estimated to be >20% among HIV‐infected patients presenting with respiratory symptoms.^68^, ^69^ These studies have also indicated that outcomes are poor in Sub‐Saharan Africa, with case fatalities reaching up to 60% in those admitted to intensive care units in Cape Town, South Africa.^70^ Therefore, PCP contributes substantially to the burden of disease in countries with high rates of HIV, which also face major challenges with diagnosis and treatment. Diagnosis relies on microscopic visualization of organisms, DNA detection in pulmonary specimens and bronchoscopy, all challenging in resource limited settings. Poor diagnosis complicated by co‐infection contributes significantly to mortality. Therefore, implementing an easy‐to‐use diagnostic test, establishing a clinical cohort to identify predictors of disease outcome and potential biomarkers for treatment response are crucial. The inability to culture *Pneumocystis* spp. in vitro, despite decades of research is a major limitation in identifying drug resistance and advancing our understanding of fungal pathogenesis.^71^ However, the improved molecular biologic methods and the identification of the genome create optimism for future understanding of the biology of *Pneumocystis* spp. and understanding the requirements for growth outside of the host lung.^62^, ^63^

## 4 DIMORPHIC FUNGAL PATHOGENS

An interesting and concerning group of fungal pathogens is classified as thermally dimorphic fungi. While certain species can infect healthy individuals, the majority take advantage of a weakened immune system to cause disease. Interestingly, these opportunistic pathogens are found in the environment in a mycelial phase and once they enter the host, the change in temperature drives a morphological switch to yeast phase and promotes disease. Many pathogen‐associated molecular pattens (PAMPs) are altered, lost or hidden during this shift making it easier for the organism to avoid recognition by the host's immune system. Recently, new species have been identified in Africa, which raise concern about their prevalence and ability to cause disease in immunocompromised patients. The symptoms of infection can vary from mild and undetected or develop into more serious conditions such as pneumonia, acute respiratory distress syndrome, and often results in disseminated disease.^72^ The severity of disease depends on exposure and immune status of the individual. Considering the percentage of patients in Africa with advanced HIV disease, it is understandable that these organisms have been given the opportunity to cause life‐threatening disease in these populations.

Globally, the major dimorphic pathogens include *Coccidioides* spp., *Histoplasma* spp. and *Talaromyces* spp., with Coccidiomycosis or Valley Fever, being the only CDC notifiable disease and restricted to areas of North and South America. In Latin America, Histoplasmosis is a major concern and recent research suggests that rates of infection in patients with advanced HIV disease now surpass those of tuberculosis (TB).^73^, ^74^ Histoplasmosis has similar clinical features to TB, and misdiagnosis could be more common than originally anticipated. Improved diagnosis and epidemiological data could provide a better understanding of the prevalence, particularly in Africa where the burden of TB is high. Talaromycosis is predominant in Asia, where there has been a recent call for its recognition as a neglected tropical disease, due to lack of investment for its diagnosis, management and research.^75^ Talaromyces infections seem to be limited to Asia with very few cases reported across Africa. Overall, dimorphic fungi contribute to infection in Africa, but the prevalence data are severely limited, and it is anticipated that the numbers are significantly higher than reported and that many cases may indeed be missed or misdiagnosed as TB.

For Africa, important diseases caused by thermally dimorphic pathogens include emergomycosis, histoplasmosis and blastomycosis. First described in South Africa in 2013, *Emergomyces africanus*, has quickly become the most diagnosed dimorphic fungal infection in Southern Africa.^76^ Subsequently and following a revision in the taxonomy of Ajellomycetaceae (which includes *Emergomyces* spp., *Emmonsia* spp. and *Blastomyces* spp.), five species of Emergomyces have been described spanning four continents.^77^, ^78^ Patients with emergomycosis have all been immunocompromised, including with HIV infection, solid organ transplantation, haematological malignancies and on immunosuppressant therapy. Dimorphic fungi have the unique ability to evade immune recognition, due to the change in morphology once entering the host. The closely related dimorphic fungus *Histoplasma capsulatum* has been shown to block innate immune recognition by β‐glucan receptors, thereby evading the host immune response.^79^ Here, the α‐(1,3)‐glucan present in the *Histoplasma spp*. cell wall was shown to mask β‐glucan and, therefore, block recognition by the PRR, Dectin‐1. Furthermore, the production of proinflammatory TNFα was suppressed in the presence of α‐glucan. However, very little is known about how the host responds to and clears *Emergomyces* spp., and what the factors that contribute to disease progression. Ribosomal DNA sequencing is excellent for identifying cultured isolates and organisms embedded within tissue sections. However, this technology is mostly not available in resource‐limited settings. As there are currently no sensitive or specific serological tests or biomarkers for the diagnosis of emergomycosis, most cases remain undetected.^80^ Therefore, developing a sensitive and specific rapid diagnostic test for use on non‐invasive clinical specimens is paramount to facilitate epidemiology studies to determine the full spectrum and true global burden of disease.

While Histoplasma is the most common dimorphic pathogen in South America, the true prevalence in Africa remains to be elucidated. Considerable differences have been noted between the African and North American varieties of *H. capsulatum*. Elegantly reviewed by Oladele and colleagues, the distribution of *H. capsulatum* variation *duboisii* (Hcd) was compared to *H. capsulatum variation capsulatum* (Hcc).^81^, ^82^ Essentially, Hcd or “African histoplasmosis” is restricted to Africa, whereas Hcc is found both globally and in Africa and is the most common cause of infection. Nigeria, South Africa and Zimbabwe have the highest prevalence of Histoplasmosis, which correlates with the high rates of HIV infection. Like Emergomycoses, only rapid diagnosis using microscopy is available, while antigen testing and PCR are very limited throughout Africa. The use of the Histoplasma EIA designed to detect antigen from urine specimens of patients with disseminated disease or the RT‐qPCR test to distinguish Histoplasma from Emergomyces would be beneficial in diagnosis and facilitating epidemiological studies. While these tests have been developed, the availability in Africa is severely limited. Furthermore, treatment options are limited, with first line therapy including Amphotericin B and Itraconazole being unlicenced or unavailable in many African countries.

Other dimorphic pathogens have been described in Africa but are less common, such as outbreaks of sporotrichosis in mine workers^83^ or the first reported case of disseminated coccidioidomycosis in Uganda.^84^ However, an interesting recent paper by Maphanga et al. describes a new species of *Blastomyces* in Africa.^85^ In light of new technologies to evaluate dimorphic fungal strains and the discovery of a novel species, *E. africanus*,^76^, ^86^ the authors revaluated 20 cases of what was previously identified as *Blastomyces dermatitidis*.^85^ Following whole genome sequencing, the South African cases of blastomycosis were found to be caused by two species distinct from *B. dermatitidis*. These were the recently described *B. percursus* and a new species named *B. emzantzi*.^85^ Initially, the *Blastomyces* genus was thought to comprise a single species, but the previous discovery of three new species and now this new species found in South Africa suggests a more detailed analysis may be required which will lead to the identification of new and emerging strains across Africa and globally. The emergence of new dimorphic fungal pathogens associated with advanced HIV disease highlights the importance of implementing easy‐to‐use diagnostic tests and creating awareness, particularly in resource limited settings as this would have important public health implication. Furthermore, a better understanding of the host immune response and, therefore, the development of personalized medicine with the use of host‐directed therapy would promote patient survival from these neglected and emerging pathogens.

## 5 *CANDIDA* SPP.

Defects in host immunity leading to serious candida infections are mainly acquired; incidence is greatly influenced by use of immunosuppressive therapy and access to other iatrogenic risk factors such as ICU care, organ transplantation, and chemotherapy. Consequently, with more widespread use of these interventions, invasive candidiasis has become an important nosocomial infection globally, associated with high morbidity and mortality. *C. albicans* is the leading cause of invasive fungal infection in the Northern Hemisphere with mortality rates approaching 40%^87^ and *Candida* spp. are among the top five pathogens recovered nosocomial bloodstream infections, particularly in high‐income countries.^88^ In a recent survey, the estimated incidence of candidemia in the United States was 7.0 per 100,000 people^89^ with similar rates in European countries, although with variation across geographic and clinical settings.^87^, ^90^, ^91^

Incidence of invasive candidiasis in Africa is unknown. Institution‐based studies provide some insights, but small numbers and lack of consistent incidence denominators preclude direct comparisons with other settings. A single hospital study in Soweto, South Africa, which described 266 cases of candidemia between 1998 to 2007, estimated an incidence of 3.6 episodes per 10,000 admissions,^92^ much lower than high income settings. Based on clinical experience, the epidemiology of candidiasis in sub‐Saharan Africa has been driven by HIV which, because of CD4 T‐cell dysfunction, is dominated by mucocutaneous forms but is rarely associated with invasive disease in the absence of additional risk factors.^93^ Another possible explanation for an apparently low burden of invasive candidiasis in Africa is limited availability of medical interventions that increase risk, such as organ transplantation. However, the combination of inaccurate diagnostic tests (sensitivity of blood culture is <50% for deep‐seated candidiasis^94^, ^95^), limited access to microbiology services and imaging, and absence of systematic reporting may lead to genuine underestimates in these resource‐limited settings.

Scale up of antiretroviral therapy has led to massive reductions in opportunistic diseases, including severe mucocutaneous candidiasis.^96^, ^97^ There may now be a shift toward invasive candidiasis, particularly in low‐middle income countries (LMICs), such as South Africa, which has a large private healthcare sector and expanding access to medical services that increase candidiasis risk. Illustrating this, in the Soweto study described, above 19% patients with candidemia were HIV‐positive,^92^ and in a more recent national survey, the incidence of candidemia was estimated at 83 cases per 100,000 admissions across 133 South African hospitals. Mirroring experience from high income countries,^89^, ^98^, ^99^, ^100^ there is evidence that this shift is accompanied by an increasing proportion of non‐albicans *Candida* infection with reduced azole susceptibility.^90^ Clinical studies repeatedly document an association between fluconazole exposure and candidemia caused by non‐*albicans* species in ICU patients and in stem cell transplantation.^101^, ^102^ Expanded access to organ transplantation and cancer therapy has led to more widespread and prolonged use of prophylactic antifungal agents, especially azoles which are fungistatic, driving selection of resistant *Candida* species.^103^ Extensive use of fluconazole for HIV‐associated cryptococcal disease in Sub‐Saharan Africa could also contribute to development of *Candida* heteroresistance and treatment failure within individuals, and theoretically to increasing population‐level azole resistance.^104^ Supporting this epidemiological shift, a laboratory‐based surveillance study in South Africa found that while *C. albicans* still predominated in public sector facilities (46%), most isolates from candidemia episodes in private sector hospitals were non‐albicans species dominated by *C. parapsilosis*, and only 37% of which were susceptible to both fluconazole and voriconazole.^105^ Alarmingly, more recent data suggest that *C. auris*, an emerging multidrug resistant and highly pathogenic nosocomial pathogen, is driving an epidemic of non‐albicans candidemia in South African private hospitals^106^, ^107^; it has now been identified in almost 100 hospitals in that country.^108^ *C. auris* has only been reported from one other African country in a single‐center study from Kenya where it was reportedly the most common cause of candidemia, accounting for 45 (38%) episodes.^109^

*C. auris* is distinguished by specific resistance mutations in the *ERG11* gene with almost universal resistance to fluconazole, often co‐existing with reduced susceptibility to other azoles and antifungal classes,^110^, ^111^, ^112^ and a proclivity for healthcare‐associated outbreaks.^113^, ^114^ *C. auris* possesses similar virulence factors to *C. albicans*, explaining its ability to cause invasive disease in at‐risk patients.^111^, ^114^ The high mortality rates associated with invasive *C. auris* infections are consistent with a generally ill patient population, and attributable mortality is unknown. However, potential for increased virulence was demonstrated in a zebrafish model where *C. auris* infection stimulated genes involved in proinflammatory cytokine pathways and was associated with downregulation of genes relating to leukocyte recruitment.^115^

Genomic studies suggest near‐simultaneous independent emergence of *C. auris* on multiple continents followed by local clonal expansion, partially explaining its insidious spread and sudden global appearance.^112^, ^114^, ^116^ Misidentification as *Candida haemulonii* and *Rhodotorula glutinis* by commercial identification systems used in most diagnostic laboratories^111^ (many of which do not routinely perform species‐level identification for Candida in resource‐limited countries) is another key factor in delayed *C. auris* recognition. Invasive infection and colonization occur almost exclusively in patients in high‐dependency areas with extensive medical intervention, and most patients with invasive *C. auris* infection have had prior exposure to antifungal agents. In this context, it is unsurprising that the only reports of *C. auris* from Sub‐Saharan Africa are from two countries with relatively advanced healthcare systems providing access to antifungals and fungal identification methods. There is a high probability that *C. auris* is already established in other African LMICs but evades detection in the absence of active surveillance. As disease profiles shift and access to health services improve with continued economic development, invasive candidiasis, and specifically *C. auris*, presents an emerging threat to human health in Sub‐Saharan Africa. Establishment of microbiological and surveillance systems, along with antifungal stewardship programs, is needed to avert this incipient public health challenge in Africa.^117^

## 6 INVASIVE FUNGAL DISEASES ASSOCIATED WITH COVID‐19

Several opportunistic invasive fungal diseases (IFDs) have been described in association with severe Covid‐19. The most prominent is invasive pulmonary aspergillosis (IPA), with prevalence ranging from 5%–30%, depending on case definition and setting.^118^, ^119^ A large French cohort study (*n* = 565) that systematically investigated Covid‐19‐associated pulmonary aspergillosis (CAPA) reported a prevalence of 15% for proven or probable IPA among mechanically ventilated patients; those with IPA had an almost 2‐fold increased risk of mortality.^120^ In other settings, excess mortality from CAPA is up to 25% compared with patients without IPA and overall mortality of CAPA is up to 50%.^119^, ^121^, ^122^

Predisposition for IPA in severe Covid‐19 disease is multifactorial. Respiratory viral infections, including influenza and SARS‐CoV‐2, the causative agent of Covid‐19, impair mucociliary clearance and cause direct damage to airway epithelium and type II pneumocytes, enabling tissue invasion by aspergillus.^123^, ^124^, ^125^, ^126^ Diffuse alveolar damage and development of ARDS, a known association with IPA in other settings, may also contribute.^127^ Perturbations of host immunity specifically pro‐inflammatory cytokine (IL‐6, IL‐1) responses leading to tissue and vascular injury and inhibition of type III interferon may promote hyphal invasion and IPA pathogenesis.^118^, ^128^, ^129^ Another putative mechanism is activation of danger pathway signalling in response to virally mediated cell death that has also been implicated in aspergillosis pathogenesis.^125^, ^127^, ^130^ Lymphopenia, frequently associated with severe Covid‐19, is a known risk factor for IPA in haematological malignancy.^131^ Immunomodulatory agents commonly used in the treatment of Covid‐19 pneumonia, including corticosteroids, IL‐6 inhibitors, and janus kinase (JAK) inhibitors, have the potential to increase risk of CAPA (and other IFDs) through cytopenias, inhibition of cell signalling and impaired function of immune cells.^120^, ^127^, ^132^ Concomitant use of broad‐spectrum antibiotics among patients with severe Covid‐19, which is a common practice, may lead to perturbations of respiratory and gut microbiota, further predisposing to fungal invasion.^133^, ^134^ Host factors for severe Covid‐19 and IPA, such as diabetes and underlying structural lung disease, frequently coexist.^120^

Is aspergillosis an emerging fungal threat in Africa? Although Covid‐19 has reportedly caused less severe epidemics on the continent, potentially mitigating risk of CAPA, this may be a consequence of under‐reporting, and LMICs like South Africa were badly affected. Under‐ascertainment of global CAPA incidence is possible because of standard diagnostic criteria for IPA may not apply in the absence of conventional host risk factors and clinical features, which are based on patients with prolonged neutropenia or critical illness.^135^, ^136^ Although consensus definitions for CAPA have been developed, these require diagnostic tools such as CT scans, bronchoscopy and microbiological systems that are not widely available in resource‐limited environments, exacerbating the problem of under‐ascertainment in Africa.^124^ However, autopsy studies from non‐African countries suggest a much lower incidence of IFDs in Covid‐19 compared with cohort studies, raising questions about clinical relevance.^137^ Other mitigating factors for CAPA in Africa include limited access to immunomodulatory drugs (besides corticosteroids) and mechanical ventilation, interventions that are almost universally associated with CAPA.

The overall burden of aspergillosis in Africa is poorly understood but may be increasing.^138^ Tuberculosis and chronic obstructive pulmonary disease (COPD), established risk factors for invasive and chronic pulmonary aspergillosis, are common.^124^, ^134^, ^137^, ^138^ There is also increasing recognition of asthma causing substantial morbidity on the continent, with some small studies showing high prevalence of fungal sensitisation suggesting fungal asthma may be an unrecognized clinical problem.^134^, ^139^, ^140^, ^141^, ^142^ Invasive pulmonary aspergillosis is not a prominent opportunistic infection in HIV; however, there is overlap with chronic pulmonary aspergillosis syndromes because of the high prevalence of TB co‐infection in Sub‐Saharan Africa, which is an important predisposing factor for aspergillosis.^143^, ^144^ Like other IFDs, such as candidiasis and PCP, incidence of IPA may increase together with ongoing economic development as access to risk‐associated medical interventions and diagnostic testing improves in LMICs.^134^, ^142^ Of concern, triazole‐resistant *A. fumigatus* has been detected in environmental surveys in African countries.^145^ Although currently at low levels, this serves as an early warning to improve surveillance, diagnostics and antifungal stewardship on the continent.

Severe Covid‐19‐induced lung damage and use of corticosteroids predispose to non‐aspergillus mould infections, especially in immunocompromised patients. Hundreds of cases of Covid‐19‐associated mucormycosis have been reported, mostly emanating from India, which also has the highest global prevalence in the pre‐Covid‐19 era.^146^, ^147^, ^148^ Uncontrolled diabetes is almost universal, as is corticosteroid use, both conventional risk factors for mucormycosis.^147^ Severe Covid‐19 exacerbates the inflammatory state induced by hyperglycaemia, possibly potentiating risk for invasive Mucorales infection in patients with these overlapping conditions.^130^, ^149^ There are no published reports of Covid‐19‐associated mucormycosis from Africa.^148^ Difficulties in diagnosis will contribute to under‐reporting, but the incidence of Covid‐19‐associated mucormycosis is likely to reflect the underlying epidemiology of mucormycosis rather than represent an emerging clinical entity.

**FIGURE 2:**
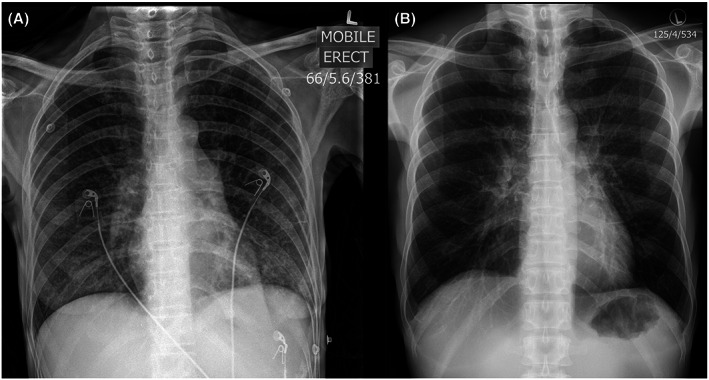
(A) A 39‐year‐old woman, HIV‐positive, failing antiretroviral therapy: CD4 count 105 cells/mm^3^, HIV viral load log 5 copies/ml. Presented with weight loss and night sweats, worsening dyspnoea, myalgia, and diarrhoea over prior few days. Started on empiric treatment for PCP which was discontinued when Covid‐19 diagnosis confirmed by PCR the day after admission. There was a good response to corticosteroids and supportive management. Chest x‐ray shows bilateral interstitial infiltrates with lower zone ground glass opacification. (B) 51‐year‐old man, newly diagnosed HIV with CD4 count 49 cells/mm^3^. *P. jirovecii* confirmed by immunofluorescent staining of induced sputum. Initial chest x‐ray shows perihilar interstitial infiltrates with areas of confluence.

Lymphopenia and use of corticosteroids in severe Covid‐19 may increase risk for concomitant *P. jirovecii* pneumonia (PCP). There have been several case reports of PCP/Covid‐19 coinfection in otherwise immunocompetent patients,^150^, ^151^ but this has not materialized as a clinically important phenomenon.^152^ More relevant is the well‐described overlap of clinical presentation and radiological features in Covid‐19 pneumonia and PCP (Figure 2).^127^ Failure to consider PCP in HIV‐positive patients (and those with other predisposing conditions such as renal transplantation) may lead to under‐diagnosis during periods of surging Covid‐19 case numbers, particularly in Sub‐Saharan African countries with high HIV burden.^153^, ^154^

## 7 FUTURE PERSPECTIVES

As an increasing proportion of people with advanced HIV disease have ART failure compounded by a surge in resistance to first‐line ART medicines, preventing opportunistic fungal infection is very important to improve patient outcome.^155^ Highlighted at the third AIDS‐related mycoses workshop in Cape Town in 2019, concerted effort has been made to address many of the challenges faced in developing countries when trying to combat these debilitating infections.^156^ This includes access to current medicines such as flucytosine,^157^ promising new clinical trial data, new diagnostic tests for neglected pathogens such as Histoplasmosis^158^ and improved advocacy. Despite the progress made, most countries in Sub‐Saharan Africa lack access to important diagnostic tests, new treatment strategies and awareness of the risks of opportunistic fungal infection. Developing easy‐to‐use, affordable, rapid diagnostic tests, like the CrAg LFA for cryptococcosis is essential for screen and treat approaches to prevent infection in at risk patients. Cryptococcal meningitis and *P. jirovecii* pneumonia contribute significantly to these infections and associated mortality.

The major issues driving the burden and impact of cryptococcal meningitis in Africa are centred around neglect of the disease in both policy and research. Like many other infectious diseases on the African continent, cryptococcosis is a disease driven by poverty and ineffective health policies. Urgent investment is required in building the capacity and quality of health care in Africa to reduce the morbidity and mortality due to cryptococcosis. Policymakers should urgently address policies surrounding drug availability and drug registration to ensure that effective antifungal drugs are available and accessible to affected populations. From a biological perspective, our understanding of the immunopathological mechanisms underlying neurocryptococcosis is still very limited. There remain huge gaps in our knowledge of how *C. neoformans* behaves after it crosses into the brain, how the brain immune cells respond to the fungus, and the molecular signalling pathways activated during infection. Additionally, there are limited translational models for studying mechanisms underlying the development of C‐IRIS. Future research could focus on developing better translational models for studying the host‐fungus interactions during cryptococcosis and C‐IRIS, especially at the cellular and molecular levels. A better understanding of host‐fungus interactions and molecular mechanisms could inform the development of more effective targeted immunotherapies and antifungal drugs.

A major barrier in reducing deaths from PCP is the availability of rapid diagnostics. Misdiagnosis leads to poor patient outcome in a population with high HIV‐TB burden; therefore evaluating new and existing diagnostic tools to improve use is imperative. Furthermore, the pathophysiology of disease is extrapolated from mouse models of infection. A more extensive understanding of host‐pathogen interactions in patients could assist with the development of these diagnostic tools, could lead to the identification of biomarkers associated with risk and potentially identify targets for personalized medication in a host‐directed therapy approach. Currently, there is a lack of genetic and other biomarkers that would allow identification of these high‐risk individuals and establishing a cohort of infected patients would greatly advance knowledge in this area. One of the greatest challenges in *Pneumocystis* research is the lack of a viable culture system. This impedes the development of better treatment options, understanding drug interactions particularly with ART, and in vitro assays that would clarify host/pathogen interactions. Recently discussed by Cushion et al, finding new approaches to identify a viable culture system for *Pneumocystis* would revolutionize the approach to combating PCP.^71^ Now that the genome has been published important information is available on how *Pneumocystis* uses host factors for survival.^62^ Using advanced modelling systems, potential supplements can be identified to develop a growth system. The development of tools mentioned above would have an immediate impact on mortality rates and would also facilitate the collection of better‐defined and accurate epidemiological data.

Fortunately, severe Covid‐19 pneumonia is now infrequent with improved access to effective Covid‐19 vaccination and high levels of population immunity from natural infection, making CAPA and other Covid‐19‐associated IFD less important clinical entities.

While not widely recognized as a threat, the risk posed by dimorphic fungal pathogens is underestimated. With organisms such as *E. africanus* being the most prevalent dimorphic fungus in South Africa, and Histoplasmosis surpassing tuberculosis in certain South American countries, it is equally important that investment is made to reduce burden and improve clinical outcome for these infections. Improved diagnostics and availability of these tests in resource poor settings are imperative. Advocacy for better access to safe and effective therapy (e.g., liposomal amphotericin B) and dedicated funding programmes for mycology research in Africa is needed to tackle emerging fungal threats.

## FUNDING INFORMATION

This work was supported by the Wellcome Trust through core funding from the Wellcome Centre for Infectious Diseases Research in Africa (203135/Z/16/Z). Sean Wasserman was supported by the National Institutes of Health (K43TW011421). Jennifer Claire Hoving is supported by the Wellcome Trust (209293/Z17/Z). For the purpose of Open Access, the author has applied a CC BY public copyright licence to any Author Accepted Manuscript version arising from this submission.

## CONFLICT OF INTEREST

All authors declare no conflict of interest.

8

### PEER REVIEW

The peer review history for this article is available at https://publons.com/publon/10.1111/pim.12953.
